# Mechanism of Cellular Formation and In Vivo Seeding Effects of Hexameric β-Amyloid Assemblies

**DOI:** 10.1007/s12035-021-02567-8

**Published:** 2021-10-04

**Authors:** Céline Vrancx, Devkee M. Vadukul, Nuria Suelves, Sabrina Contino, Ludovic D’Auria, Florian Perrin, Vincent van Pesch, Bernard Hanseeuw, Loïc Quinton, Pascal Kienlen-Campard

**Affiliations:** 1 Alzheimer Research Group, Cellular and Molecular Division (CEMO), Institute of Neuroscience, Université Catholique de Louvain, 1200 Brussels, Belgium; 2 Neurochemistry Unit, Cellular and Molecular Division (CEMO), Institute of Neuroscience, Université Catholique de Louvain, 1200 Brussels, Belgium; 3 Department of Neurology, Cliniques Universitaires Saint-Luc, Université Catholique de Louvain, 1200 Brussels, Belgium; 4 Laboratory of Mass Spectrometry, Department of Chemistry, Université de Liège, 4000 Liège, Belgium

**Keywords:** Alzheimer’s disease, Aβ oligomers, Hexameric Aβ, Presenilins, FAD model, Seeding

## Abstract

The β-amyloid peptide (Aβ) is found as amyloid fibrils in senile plaques, a typical hallmark of Alzheimer’s disease (AD). However, intermediate soluble oligomers of Aβ are now recognized as initiators of the pathogenic cascade leading to AD. Studies using recombinant Aβ have shown that hexameric Aβ in particular acts as a critical nucleus for Aβ self-assembly. We recently isolated hexameric Aβ assemblies from a cellular model, and demonstrated their ability to enhance Aβ aggregation in vitro. Here, we report the presence of similar hexameric-like Aβ assemblies across several cellular models, including neuronal-like cell lines. In order to better understand how they are produced in a cellular context, we investigated the role of presenilin-1 (PS1) and presenilin-2 (PS2) in their formation. PS1 and PS2 are the catalytic subunits of the γ-secretase complex that generates Aβ. Using CRISPR-Cas9 to *knockdown* each of the two presenilins in neuronal-like cell lines, we observed a direct link between the PS2-dependent processing pathway and the release of hexameric-like Aβ assemblies in extracellular vesicles. Further, we assessed the contribution of hexameric Aβ to the development of amyloid pathology. We report the early presence of hexameric-like Aβ assemblies in both transgenic mice brains exhibiting human Aβ pathology and in the cerebrospinal fluid of AD patients, suggesting hexameric Aβ as a potential early AD biomarker. Finally, cell-derived hexameric Aβ was found to seed other human Aβ forms, resulting in the aggravation of amyloid deposition in vivo and neuronal toxicity in vitro.

**Supplementary Information:**

The online version contains supplementary material available at 10.1007/s12035-021-02567-8.

## Introduction

The β-amyloid peptide (Aβ) is the major constituent of senile plaques, a typical histological hallmark of Alzheimer's disease (AD). This peptide is produced by the amyloidogenic processing of the amyloid precursor protein (APP) [1]. APP undergoes a first cleavage by β-secretase, producing a 99-amino acid membrane-bound C-terminal fragment (βCTF or C99), which in turn is cleaved by γ-secretase to generate the intracellular domain of APP (AICD) and Aβ. To note, different isoforms of Aβ can be produced, mostly ranging from 38 to 43 amino acids [2]. After its release as a monomer, Aβ and particularly its longer forms such as Aβ_42_, has the propensity to self-assemble [3]. This leads to the formation of Aβ oligomers and ultimately amyloid fibrils, aggregating into senile plaques in the brain.

Many studies suggest that the toxicity of Aβ is not related to insoluble fibrils but rather to soluble oligomeric intermediates generated during fibrillogenesis. Such a toxic property for Aβ oligomers may result from their intrinsically misfolded nature and their aggregation propensity that can together contribute to trap vital proteins or cause cell membrane alterations [4, 5]. In addition, it is believed that the stability of Aβ, and particularly of Aβ oligomers, gives them persistent and aggravating pathological properties for the formation of amyloid deposits [6, 7].

Several oligomeric Aβ species have been suggested to play an important role in the process of Aβ self-assembly. Among them, Aβ hexamers gain increasing interest. They are smallest assembly readily formed by synthetic Aβ_42_ in solution [3, 8]. Aβ assembly relies on a process of nucleated polymerization [9, 10], involving a nucleation phase where Aβ monomers self-associate to form an oligomeric nucleus. Findings from structural studies indicate that hexameric Aβ assemblies might behave as such nuclei, serving as a building block for the formation of higher assemblies [11–13]. However, the cellular context leading to the formation of hexameric Aβ assemblies is still poorly understood.

We previously reported the presence of ~ 28 kDa Aβ assemblies in Chinese hamster ovary (CHO) cells expressing amyloidogenic fragments of human APP [14]. Biochemical approaches demonstrated that these assemblies likely correspond to Aβ_42_ hexamers [15]. Here, we report the identification of similar, hexameric-like, Aβ assemblies across several cell lines, including a neuronal-like cell line. We studied the cellular pathway that potentially contributes to hexameric Aβ formation and propagation. The production of Aβ in a cellular context relies on the γ-secretase activity. The catalytic core of the γ-secretase complex is formed by either the presenilin-1 (PS1) or the presenilin-2 (PS2) protein [16]. PS1 has been repeatedly reported as the major contributor to Aβ production [17–21], rendering the role of PS2 in amyloid pathology less understood. Recent findings revealed the enrichment of PS2 γ-secretases in endosomal compartments [22]. A lower contribution of PS2 to overall Aβ production could thus be explained by a secondary encountering of substrates along cellular trafficking. Importantly, PS2 γ-secretases were shown to generate mostly an intracellular pool of Aβ and to favor the accumulation of aggregation-prone Aβ_42_ in endocytic compartments [23]. Based on these observations, we aimed at discriminating the contribution of PS1 and PS2 γ-secretases to the production of the Aβ assemblies we identified. We generated human neuronal-like cell lines *knockdown* for each of the two presenilins and provide evidence for a specific correlation between the PS2-dependent γ-secretase and the vesicular release of hexameric-like Aβ assemblies. This suggests a key role for the γ-secretase present in the late endosomal/lysosomal compartments both in the production and in the mode of release of Aβ oligomers.

As different species of Aβ oligomers were suggested to exert neurotoxic effects [24–28], a crucial point was then to understand if the identified hexameric-like Aβ assemblies produced and released by cells readily appeared in pathological conditions, and if they displayed toxic properties. We found hexameric-like Aβ to be present in the 5xFAD mouse model of amyloid pathology [29] and the cerebrospinal fluid of human AD patients. This reinforces the role of Aβ hexamers in pathological conditions. We further assessed the ability of hexameric Aβ isolated from cells to drive amyloid deposition and induce neuronal toxicity. To this end, we used two mouse models: control wild-type mice (C57BL/6) to measure the ability of the hexamers to form amyloid deposits in a non-pathological context, and transgenic 5xFAD mice to study their effect in an environment where Aβ is pre-existing. We found that cell-derived hexameric Aβ does not induce toxic effects by itself, but enhances Aβ deposition in a pathological context where human Aβ accumulates (5xFAD).

## Materials and Methods

### Chemicals and Reagents

Reagents used for Western blotting—Pierce BCA protein assay kit, SeeBlue™ Plus2 pre-stained standard, NuPAGE™ 4–12% Bis–Tris protein gels, NuPAGE™ MES SDS Running Buffer (20 ×), NuPAGE™ Transfer Buffer (20 ×), nitrocellulose 0.1 μm membranes, and GE Healthcare ECL Amersham™ Hyperfilm™—were all purchased from ThermoFisher (Waltham, MA, USA). Western Lightning® Plus-ECL was from PerkinElmer (Waltham, MA, USA). Complete™ protease inhibitor cocktail was from Roche (Basel, Switzerland). Primary antibodies targeting human Aβ: anti-Aβ clone W0-2 (MABN10), anti-Aβ_40_ clone 11A5-B10 (05-799), and anti-Aβ_42_ clone 12F4 (05-831-I) were from Merck (Kenilworth, MJ, USA). Anti-PS1 (D39D1) and anti-PS2 (D30G3) antibodies were from Cell Signaling (Danvers, MA, USA). Anti-APP-Cter (A8717) and anti-α-tubulin primary antibodies as well as secondary antibodies coupled to horseradish peroxidase (HRP) were obtained from Sigma-Aldrich (St-Louis, MO, USA). Alexa Fluor™ 647 secondary antibody was obtained from ThermoFisher. Thioflavin T (ThT) amyloid stain was obtained from Sigma-Aldrich. Mowiol® 4-88 used for mounting medium was purchased from Merck. Cell culture reagents—Ham’s-F12, DMEM-F12, DMEM, and Neurobasal® growth media, penicillin–streptomycin (p-s) cocktail, Lipo2000® transfection reagent, Opti-MEM®, HBSS, glutamine and B-27®—were all purchased from ThermoFisher. Fetal bovine serum (FBS) was from VWR (Radnor, PA, USA). GELFrEE™ 8100 12% Tris–Acetate cartridge kits were purchased from Expedeon (Heidelberg, Germany). ReadyProbes® cell viability assay kit was from ThermoFisher. ELISA strip plates for immuno-Europium assay (F8, high-binding 771261) were from Greiner Bio-One (Frickenhausen, Germany) and reagent diluent-2 10 × (DY995) from R&D systems (Minneapolis, MN, USA). Anti-CD9 primary antibody (MAB1880) was from R&D systems, anti-CD81 (TAPA-1, 349502) from BioLegend (San Diego, CA, USA), anti-CD63 (MCA2142) from Serotec Bio-Rad (Kidlington, UK), and anti-GM130 (610823) from BD transduction (Franklin Lakes, NJ, USA). The anti-mouse IgG-biotin (NEF8232001EA), Europium-labeled streptavidin (1244–360), Delfia® wash concentrate 25 × (4010–0010), Delfia® assay buffer (1244-111), and Delfia® enhancement solution (1244-105) were all from PerkinElmer.

### DNA Constructs

The pSVK3-empty (EP), -C42 and -C99 vectors used for expression in rodent cell lines (CHO, MEF) were described previously [14, 30]. C42 and C99 are composed of the APP signal peptide fused to the human Aβ_42_ and βCTF sequences, respectively. For the expression in human cell lines (HEK293, SH-SY5Y), the C99 construct in a pCDNA3.1 plasmid was kindly provided by R. Pardossi-Piquard (University of Sophia Antipolis, Nice, France). The pCDNA3.1 plasmid bearing the C99-GVP construct used in reporter gene assays was a gift from H. Karlström (Karolinska Institute, Stockholm, Sweden). The associated Gal4RE-*Firefly* luciferase reporter gene (pG5E1B-luc) and *Renilla* luciferase reporter vector (pRL-TK) have been described previously [21, 31, 32].

### Cell Lines Culture and Transfection

Chinese hamster ovary (CHO) cell lines were grown in Ham’s-F12 medium; Human neuroblastoma SH-SY5Y cells and mouse embryonic fibroblasts (MEF) in DMEM-F12; Human embryonic kidney cells (HEK293 and HEK293-T) in DMEM. All media were supplemented with 10% of heat-inactivated FBS and 100units/ml p-s. All cell cultures were maintained at 37 °C in a humidified atmosphere and 5% CO_2_.

For transient transfection, 40,000cells/cm^2^ were seeded 24 h before transfection. Transfection mixes containing desired DNA and Lipo2000® were prepared in Opti-MEM® and pre-incubated for 15 min at room temperature (rt). One day after transfection, medium was changed to fresh FBS-free culture medium and incubated for another 24 h. Cell lysates and culture media were harvested 48 h after transfection for analysis.

### Western Blotting

Cells were rinsed and scraped in phosphate-buffered saline (PBS) and centrifuged for 5 min at 7000×*g*. Pellets were sonicated in lysis buffer (125 mM Tris pH 6.8, 20% glycerol, 4% SDS) with Complete™ protease inhibitor cocktail. Protein concentration was determined using the Pierce BCA protein assay kit. Proteins were heated for 10 min at 70 °C in loading buffer (lysis buffer supplemented with 50 mM dithiothreitol (DTT) and NuPAGE™ LDS sample buffer (ThermoFisher)). Samples were loaded and separated by SDS-PAGE electrophoresis on Nupage™ 4–12% Bis–Tris gels with MES SDS running buffer, using SeeBlue™ Plus2 pre-stained as a standard. Proteins were then transferred for 2 h at 30 V with NuPAGE™ transfer buffer onto 0.1 μm nitrocellulose membranes. After blocking (5% non-fat milk in PBS-Tween®20 0.1%), membranes were incubated overnight at 4 °C with the primary antibodies, then washed and incubated with the secondary antibodies coupled to HRP for 1 h prior to ECL detection. Primary antibodies were used as follows: anti-human Aβ clone W0-2 (1:1500), anti-APP-C-ter (1:2000), anti-PS1 (1:1000), anti-PS2 (1:1000), and anti-α-tubulin (1:3000). Secondary antibodies were used as follows: HRP-coupled anti-mouse IgG (1:10,000) or anti-rabbit IgG (1:10,000).

### GELFrEE™ Isolation of Cell-Derived Hexameric Aβ

**Fig. 1:**
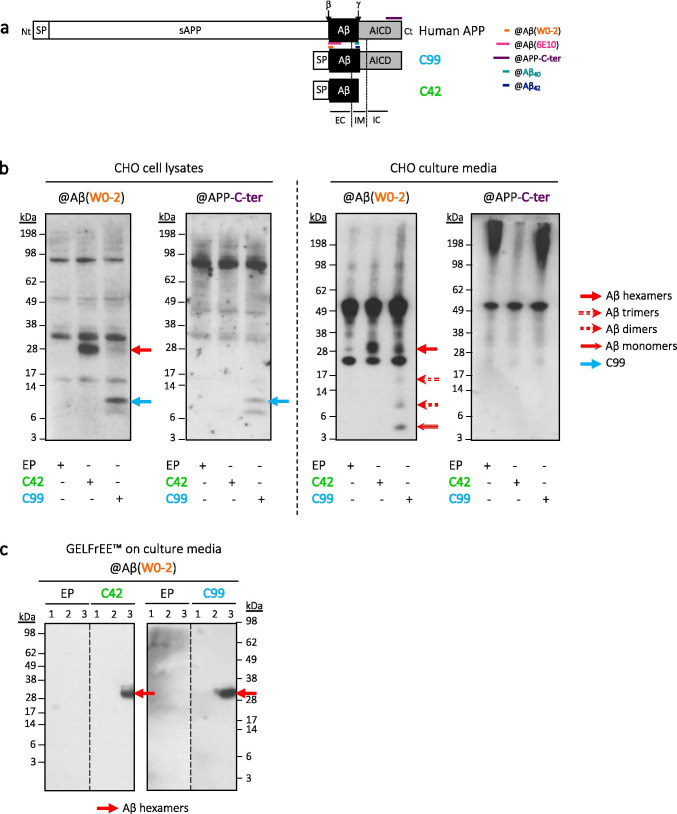
Hexameric Aβ_42_ derived from CHO cells expressing human APP metabolites. **a** Full-length APP is cleaved by β-secretase at the β site, located at the N-terminus of Aβ, to produce a 99-amino acid membrane-bound fragment (C99) encompassing Aβ and AICD. The C99 construct expressed here in the cells was fused to the signal peptide (SP) of APP. C99 is cleaved by γ-secretase to release Aβ. The C42 construct is composed of the SP of APP and the Aβ_42_ sequence. The epitopes of the primary antibodies used in this study are indicated on the scheme; either directed against human Aβ (clones W0-2 and 6E10 targeting its N-terminal part, and Aβ_40_ and Aβ_42_ antibodies specifically targeting the free C-terminal end of Aβ) or against the C-terminal region of APP (APP-C-ter). *Nt* N-terminus, *Ct* C-terminus, *sAPP* soluble APP, *AICD* APP intracellular domain, *EC* extracellular, *IM* intramembrane, *IC* intracellular. **b** Detection of ~ 28 kDa assemblies by Western blotting in CHO cell lysates and culture media following expression of either C42 or C99. These assemblies are recognized by Aβ specific antibodies (such as W0-2 here), but not by the anti-APP-C-ter, suggesting they emerge by assembly of Aβ. Media samples were lyophilized and all samples were immunoprecipitated with the anti-Aβ (W0-2) antibody prior to analysis. In the media of C99-expressing cells, intermediate assemblies are also observed; monomers, dimers, and trimers. *EP* empty plasmid. **c** Isolation of cell-derived Aβ assemblies. The media of CHO cells expressing either EP, C42, or C99 were immunoprecipitated and separated using the GELFrEE™ technique. We optimized a method to collect the ~ 28 kDa Aβ assemblies as an isolated liquid fraction. Dashed lines indicate that proteins were run on the same gel, but lanes are not contiguous

CHO cells culture media were collected 48 h after transfection with either pSVK3-EP, -C42 or -C99, lyophilized, resuspended in ultrapure water and pre-cleared with recombinant protein A sepharose (GE Healthcare, Chicago, IL, USA). Immunoprecipitation of Aβ species was performed with the monoclonal anti-human Aβ clone W0-2 antibody. Samples were separated through a gel-eluted liquid fraction entrapment electrophoresis (GELFrEE™ 8100) system to allow the collection of the desired kDa range of proteins directly in liquid fraction. The following method was used for hexameric Aβ collection: step 1: 60 min at 50 V, step 2: 6 min at 70 V, step 3: 13 min at 85 V, and step 4: 38 min at 85 V. Fractions 1, 2, and 3 (Fig. 1c) were collected at the end of steps 2, 3, and 4, respectively. All samples were collected in the system running buffer (1X buffer: 1% HEPES, 0.01% EDTA, 0.1% SDS, and 0.1% Tris) and kept on ice. Absorbance at 280 nm of each fraction was read using a BioPhotometer® D30 (Eppendorf, Hamburg, Germany) and the concentration of the collected hexameric Aβ was calculated using the molar extinction coefficient ε_280nm_ = 1490 M^−1^ cm^−1^.

### Dot Blotting

5 µl of isolated hexameric Aβ (150 µM for isoform characterization, 15 µM for fractions evaluation prior to intracerebral injection) and 5 μl of 50 µM synthetic monomeric Aβ (mAβ) with 40 (mAβ_40_) or 42 residues (mAβ_42_) were spotted onto 0.1 µm nitrocellulose membranes and allowed to dry. Another 5 µl of sample were then spotted twice on top and dried. The membranes were boiled twice in PBS for 3 min, then blocked with 5% non-fat milk in PBS-Tween®20 0.1%, washed and incubated with primary and secondary antibodies prior to ECL detection, as described above. Primary antibodies dilutions were used as follows: anti-human Aβ clone W0-2 (1:1500), anti-Aβ_40_ (1:1000), and anti-Aβ_42_ (1:1000). Secondary antibodies were used as described for Western blotting. Synthetic mAβ_40_ and mAβ_42_ were prepared as previously described [33].

### Generation of SH-SY5Y PS1- and PS2-Deficient Cells by CRISPR-Cas9

Kits each containing guide RNA vectors that target human *PSEN1* or *PSEN2* genes, a GFP–puromycin or RFP–blasticidin donor vector, respectively, and a scrambled sequence control were obtained from Origene (CAT#: KN216443 and KN202921RB). Target sequences were flanked with specific homology sequences for the stable integration of donor sequences, based on the homology-directed repair technique [34, 35]. SH-SY5Y cells were transfected using Lipo2000® and FACS-sorted 48 h later for GFP + (PS1) or RFP + (PS2) cells, then seeded in 24w plates. Following a few days for cell-growth, a second selection was performed using puromycin (PS1) or blasticidin (PS2) at a concentration of 15 μg/ml or 30 μg/ml, respectively. Cells were then allowed to grow again and split twice before subcloning in 96w plates. PS1 and PS2 clonal populations were selected for following experiments on account of the highest gene-extinction efficiency, mirrored by the strongest decrease in protein levels. Puromycin (2.5 μg/ml) and blasticidin (7.5 μg/ml) were used for the maintenance of PS1- and PS2-deficient cells, respectively.

### Dual Luciferase Assay

SH-SY5Y cells were co-transfected with Lipo2000® in a 1:1:1 ratio with pG5E1B-luc, pRL-TK, and either a pCDNA3.1-empty plasmid (EP) or the pCDNA3.1-C99-GVP, bearing a tagged C99 to quantify the release of the APP intracellular domain (AICD). The system setup was previously described [21, 32]. Cells were rinsed with PBS 48 h after transfection and incubated with the reporter lysis buffer (Promega, Madison, WI, USA) for 15 min at rt. *Firefly* and *Renilla* luciferase activities were measured using the Dual-Glo® luciferase assay system (Promega, Madison, WI, USA) on a Sirius single tube luminometer (Berthold, Bad Wildbad, Germany). Luciferase activity corrected for transfection efficiency was calculated as the *Firefly*/*Renilla* ratio.

### Electro-Chemiluminescence Immunoassay (ECLIA) for Monomeric Aβ Quantification

Aβ monomeric peptides were quantified in human CSF or in SH-SY5Y cells media using the human Aβ 6E10 multiplex ECLIA assay (Meso Scale Discovery, Gaithersburg, MD, USA) as previously described [36]. For SH-SY5Y, cells were conditioned in FBS-free medium for 24 h. After collection, medium was lyophilized and resuspended in ultrapure water prior to analysis.

### Extracellular Vesicles Isolation

Culture medium was collected and underwent several centrifugation steps (all at 4 °C): 300×*g* for 10 min for the elimination of living cells, 1000×*g* for 10 min to discard dead cells, 10,000×*g* for 30 min for the removal of cellular debris, and finally 100,000×*g* for 1 h to collect extracellular vesicles (EVs) as a pellet and soluble proteins as supernatant. Soluble proteins were precipitated by incubation with 10% trichloroacetic acid (TCA) for 30 min on ice. Both EVs and soluble proteins fractions were resuspended in 500 µl of PBS for nanoparticle tracking analysis (NTA) and plate-based Europium-immunoassay. For Western blotting, a saved portion of both fractions was sonicated in lysis buffer (125 mM Tris pH 6.8, 20% glycerol, 4% SDS) with Complete™ protease inhibitor cocktail. Protein concentration was determined using the Pierce BCA protein assay kit.

### Nanoparticle Tracking Analysis (NTA)

EVs were counted in each fraction by the ZetaView® (ParticleMetrix GmbH, Inning am Ammersee, Germany), which captures Brownian motion through a laser scattering microscope combined with a video camera to obtain size distribution (50–1000 nm) and concentration. Samples were diluted 1:50 (v:v) in PBS to reach 50–200 particles/frame, corresponding to ~ 2 × 10^7^–1 × 10^8^particles/ml. Sensitivity was set to 65 and camera shutter to 100 in order to detect less than 3 particles/frame when PBS alone was injected, to assess background signal. Measurements were averaged from particles counted in 11 different positions for 2 repeated cycles with camera at medium resolution mode.

### Plate-Based Europium-Immunoassay

50 µl of EVs and soluble proteins fractions were bound to protein-binding ELISA plates. After overnight incubation at 4 °C, the rest of the experiment was performed at rt by shaking on a tilting shaker at 30 rpm. The plate was washed with Delfia® buffer (diluted to 1 × in PBS: Delfia®-W), then blocked with reagent diluent-2 (diluted to 1% BSA in PBS) for 90 min. The bound material was labeled with primary antibodies against CD9, CD81, CD63, and GM130 (1 µg/ml in reagent diluent-2) for 90 min. After three Delfia®-W washes, goat anti-mouse biotinylated antibody (1:2500 in reagent diluent-2) was added for 60 min. After another three Delfia®-W washes, Europium-conjugated streptavidin (diluted to 1:1000 in Delfia® buffer) was added for 45 min. After six final Delfia®-W washes, Delfia® enhancement solution was incubated for 15 min before measurement using time-resolved fluorometry with excitation/emission: 340/615 nm, flash energy/light exposure: high/medium, and integration lag/counting time: 400/400 µs (VICTOR® X4 multilabel plate reader, PerkinElmer).

### Generation of SH-SY5Y PS2 Rescued Cells

Wild-type human PS2 (hPS2) generated in a previously described plasmid vector [37] was subcloned in the pLenti-CMV/TO-puro lentiviral vector (plasmid 17482, Addgene). Lentiviruses production was carried out in HEK293-T cells. 48 h after transfection with the hPS2 vector, cells were harvested and centrifuged at 1500×*g* for 10 min at 4 °C. The supernatant was filtered with an Acrodisc® 0.45 μm filter (Pall, NYC, USA). Then, 1/3 (v/v) of LentiX™ Concentrator reagent (Clontech, Mountain View, USA) was added and the solution was incubated overnight. After centrifugation at 1500×*g* for 45 min at 4 °C, the pellet was resuspended in 20 μl per dish of DMEM without serum and stored at − 80 °C until use. 80 μl of concentrated viruses were used to infect 3 × 10^6^ SH-SY5Y PS2-KD cells. Selection started 72 h after infection by adding 5 μg/ml puromycin. Recombinant cell lines were further cultivated in culture medium containing 2.5 μg/ml puromycin, and the PS2 expression profile was monitored by Western blotting.

### Animal Models

Transgenic 5xFAD mice (Tg6799) harboring human *APP* and *PSEN1* transgenes were originally obtained from the Jackson Laboratory: B6SJL-Tg(*APP*SwFlLon,*PSEN*1*M146L*L286V)6799Vas/Mmjax (34840-JAX). Colonies of 5xFAD and non-transgenic (wild-type, WT) mice were generated from breeding pairs kindly provided by Pr. Jean-Pierre Brion (ULB, Brussels, Belgium). All mice were kept in the original C57BL/6 background strain. Animals were housed with a 12 h light/dark cycle and were given ad libitum access to food and water. All experiments conducted on animals were performed in compliance with protocols approved by the UCLouvain Ethical Committee for Animal Welfare (reference 2018/UCL/MD/011).

### Protein Extraction from Mouse Brain Tissues

WT and 5xFAD mice of either sex were euthanized by cervical dislocation or using CO_2_, and brains were quickly removed. The hippocampus and a portion of temporal cortex were immediately dissected on ice. Brain tissues were then homogenized by pipetting up and down with a 1000 μl pipette and sonicating in ice-cold lysis buffer (150 mM NaCl, 20 mM Tris, 1% NP40, 10% glycerol) with Complete™ protease inhibitor cocktail until homogenous. Samples were stored at − 80 °C until use. Protein concentration was determined using the Pierce BCA protein assay kit prior to analysis.

### Cerebrospinal Fluid Collection

Cerebrospinal fluid (CSF) was collected by lumbar puncture from AD patients and symptomatic controls undergoing diagnostic work-up at the Cliniques Universitaires Saint-Luc (UCL, Brussels, Belgium), both of either sex, following the international guidelines for CSF biomarker research [38]. Collected samples were directly frozen at − 80 °C until analysis and were always manipulated on ice during Western blotting and ECLIA experiments. Included patients signed an internal regulatory document, stating that residual samples used for diagnostic procedures can be used for retrospective academic studies, without any additional informed consent (ethics committee approval: 2007/10SEP/233). AD patients participated in a specific study referenced UCL-2016-121 (Eudra-CT: 2018-003473-94). In total, CSF samples from eight subjects were retrospectively monitored in this study (see Supplementary Table.S1).

### Intracerebral Stereotaxic Surgery

2-month-old WT and 5xFAD mice of either sex were deeply anesthetized by intraperitoneal injection of a mixture of ketamine (Ketamin®) (10 mg/kg) and medetomidine (Domitor®) (0.5 mg/kg), and placed in a stereotaxic apparatus (Kopf® Instruments, Tujunga, CA, USA). 2 μl of 15 μM cell-derived hexameric Aβ (C42 fraction) or control (EP fraction) were injected using a 10 μl Hamilton syringe and an automated pump (RWD®, Guangdong, China). Coordinates used for intrahippocampal injection were based on the Paxinos atlas: A/P − 1.94; L ± 2.17; D/V − 1.96; mm relative to bregma, considering a bregma–lambda distance of 4.21 mm. When the distance differed, coordinates were proportionally adjusted. 30 days after stereotaxic injection, mice were transcardially perfused with PBS and brains were post-fixed in 4% paraformaldehyde for 24 h at 4 °C.

### Immunohistofluorescence

For immunohistological analysis, free-floating coronal sections (50 μm) were generated from agarose-embedded fixed brains using a vibrating HM650V microtome (ThermoFisher), and were preserved in PBS-sodium azide 0.02% at 4 °C. Prior to immunomarking, sections were washed in PBS and subsequently blocked and permeabilized with PBS-BSA 3%-TritonX100 0.5% for 1 h at rt. Sections were then incubated with anti-human Aβ clone W0-2 (1:100) overnight at 4 °C as a marker for Aβ-containing species. After three PBS washes and incubation with goat anti-mouse IgG Alexa Fluor™ 647 secondary antibody (1:500) for 1 h at rt, slices were finally washed three times with PBS and mounted on SuperFrost® slides. Slides were then incubated with ThT (0.1 mg/ml in ethanol 50%) for 15 min at rt as a marker for fibrillar deposits. After three washes with ethanol 80% and a final wash with ultrapure water, coverslips were mounted with Mowiol® 4–88-glycerol. W0-2 and ThT staining were detected with standard FITC/Cy5 and GFP filters, respectively, at an EVOS® FL Autofluorescence microscope. Counting of double-positive dots was performed on ImageJ.

### Primary Neuronal Cultures

Primary cultures of neurons were performed on mouse embryos of either sex at embryonic day 17 (E17), as described previously [39]. Briefly, cortices and hippocampi were isolated by dissection on ice-cold HBSS and meninges were removed. Tissues were then dissociated by pipetting up and down 15 times with a glass pipette. Dissociation was repeated 10 times with a flame-narrowed glass pipette and samples were allowed to sediment for 5 min. Supernatants containing isolated neurons were then settled on 4 ml FBS and centrifuged at 1000×*g* for 10 min. Pellets were resuspended in Neurobasal® medium enriched with 1 mM L-glutamine and 2% B-27® supplement medium. 100,000cells/cm^2^ were plated in 12w plates pre-coated with poly-L-lysine (Sigma-Aldrich). Cultures were maintained at 37 °C and 5% CO_2_ in a humidified atmosphere.

### Cell Viability Assay (ReadyProbes®)

Primary neuronal cultures performed from WT and 5xFAD mouse embryos of either sex were incubated at 7 days in vitro (DIV7) with 1 or 5 µM of either cell-derived hexameric Aβ (C42 fraction) or control (EP fraction). At DIV8, 2 drops/ml of each reagent of the ReadyProbes® assay were added to cells: NucBlue® Live reagent for the staining of all nuclei and NucGreen® Dead reagent for the nuclei of cells with compromised plasma membrane integrity. Staining were detected with standard DAPI and FITC/GFP filters, respectively, at an EVOS® FL Autofluorescence microscope. Quantification was performed by counting dead vs total cells on ImageJ.

### Statistical Analyses

The number of experiments (*N*) and the number of samples per condition in each experiment (*n*) are indicated in figure legends. All statistical analyses were performed using the GraphPad Prism 8 software (GraphPad Software, La Jolla, CA, USA). All datasets were assessed for gaussian distribution using the Shapiro–Wilk test. A parametric test was applied if the data followed normal distribution. Otherwise, non-parametric tests were used. Statistical analysis performed in each case is indicated in the corresponding figure legend. Briefly, when tested groups were expressed as a fold-change of their corresponding control, the value of the control was set as the hypothetical value for the use of parametric one-sample *t* test or non-parametric one-sample Wilcoxon single-ranked test. When a correlation between two variables was assessed, Pearson’s *R* correlation coefficient was calculated. When two groups were compared, parametric *t* or non-parametric Mann–Whitney tests were used. When more than two groups were compared, parametric ANOVA with indicated post hoc tests or non-parametric Kruskal–Wallis were used. Significance is indicated as follows: ns = non-significant, *p < 0.05, **p < 0.01, ***p < 0.001. Actual p-values of each test are indicated in the corresponding figure legend.

## Results

### Identification of Hexameric Aβ_42_ Assemblies Across a Wide Range of Cell Lines

The growing body of evidence pointing to the pathological properties of oligomeric—and particularly hexameric—Aβ assemblies mostly stems from observations gathered using synthetic Aβ peptides. We focused on the identification of Aβ assemblies that are readily formed in a cellular context. These studies were first carried out in CHO (Chinese hamster ovary) cells transiently transfected with vectors expressing the human sequences of either Aβ_42_ (referred to as C42) or βCTF (C99), each fused to the APP signal peptide to ensure a proper cellular trafficking of the expressed fragments (Fig. 1a). The Aβ assemblies formed in this cellular context were analyzed by Western blotting (Fig. 1b) using a combination of antibodies, targeting epitopes present in either the human Aβ sequence (W0-2 and 6E10) or in the APP C-terminal region (APP-C-ter) (Fig. 1a). As previously reported [14, 15], we repeatedly detected a band at ~ 28 kDa in both C42 and C99-expressing cells with Aβ-specific antibodies (W0-2 in Fig. 1b and 6E10 in Supplementary Fig.S1). These assemblies were not recognized by the anti-APP-C-ter specific antibody (Fig. 1b), indicating that they are formed by self-association of the Aβ fragment and do not correspond to the previously reported C99 dimers [30, 40]. Their observed size corresponds to the theoretical size of hexameric Aβ. In addition, intermediate Aβ assemblies—likely corresponding to monomers, dimers and trimers of Aβ—were detected in the media of C99-expressing cells. As the cleavage of C99 in a cell generates various forms of Aβ and mostly Aβ_40_, this suggests that assemblies smaller than hexamers are formed when such various forms of Aβ are produced upon C99 processing, but that hexameric forms are predominant when only Aβ_42_ is produced by cells, as is the case for C42-expressing cells.

For a further characterization of the identified Aβ assemblies, we used a gel-eluted liquid fraction entrapment electrophoresis technique (GELFrEE™ 8100) to isolate the cell-derived Aβ assemblies from W0-2-immunoprecipitated media of C42 or C99-expressing CHO cells (Fig. 1c). This technique was recently used for extensive biochemical analyses of oligomeric Aβ produced by cells [15]. Dot blotting with primary antibodies directed against the free C-terminal end of the two major Aβ isoforms (Aβ_40_, Aβ_42_) was performed on the isolated ~ 28 kDa assemblies. It indicated they were composed of Aβ_42_ (see Supplementary Fig.S2 and [15]). Thus, the Aβ assemblies we identified in a cellular context likely correspond to hexameric Aβ_42_ assemblies.

**Fig. 2:**
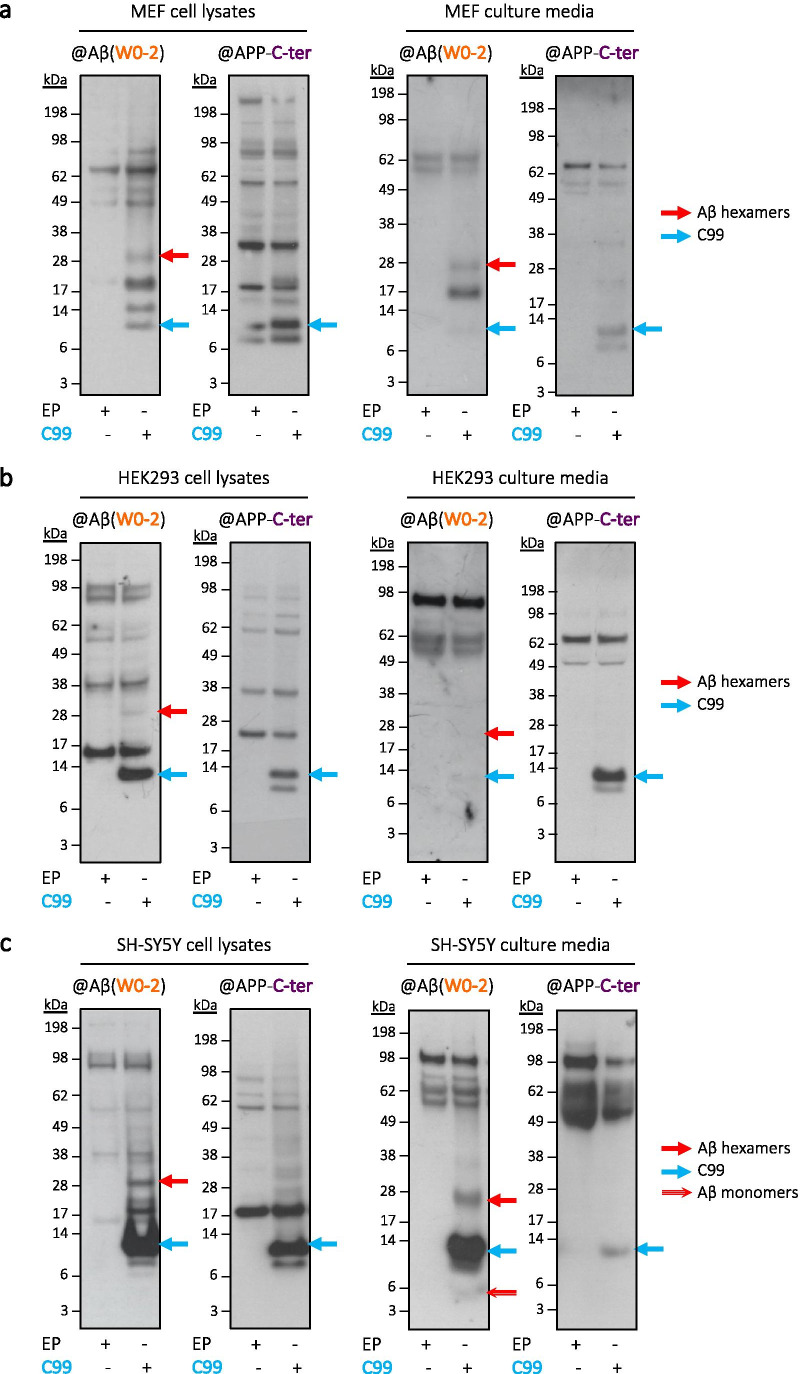
Commonality of hexameric Aβ production in several cell lines. Cell lysates and media from murine MEF fibroblasts (in **a**) and human embryonic HEK293 cells (in **b**), as well as from human neuroblastoma-derived SH-SY5Y cells (in **c**) expressing C99 all revealed the presence of ~ 28 kDa assemblies recognized by the human Aβ-specific W0-2 antibody, and not by the anti-APP-C-ter. Media samples were lyophilized prior to analysis. *EP* empty plasmid

As Aβ self-assembly strongly depends on the context of its release, we sought to determine whether the assemblies of interest were produced specifically by CHO cells or more generally across various cell lines. Using the same procedure as described above [15], we assessed the Aβ profile in transiently transfected mouse embryonic fibroblasts (MEF) (Fig. 2a) as well as in two human cell lines: human embryonic kidney (HEK293) cells (Fig. 2b) and neuroblastoma-derived SH-SY5Y cells (Fig. 2c). Importantly, the ~ 28 kDa assemblies were consistently detected with the W0-2 Aβ-specific antibody and not by the APP-C-ter-targeted antibody in all the tested models (Fig. 2). The amounts of ~ 28 kDa Aβ assemblies produced by the different cell lines are variable and notably lower in HEK293, but our results indicate that similar, hexameric-like Aβ assemblies can readily form across different cell lines and are not restricted to one cell-type, fostering their relevance as a critical cell-derived Aβ assembly.

### Cellular Pathways and Contribution of Presenilins to the Formation of Hexameric-Like Aβ Assemblies

We next aimed at understanding the cellular context in which hexameric-like Aβ is formed. In a cell, the production of any Aβ species requires the catalytic processing of the C99 fragment by the γ-secretase complex, a step that is ensured either by presenilin-1 (PS1) or presenilin-2 (PS2). Previous studies demonstrated that PS1- and PS2-dependent γ-secretases have differential substrate specificities [21, 41] and that several factors, including their specific subcellular localization [23], can promote the production of longer and more aggregation-prone Aβ isoforms. Thus, we investigated the contribution of PS1- and PS2-dependent γ-secretases to the formation of Aβ species that would be capable of assembling into hexameric-like Aβ.

**Fig. 3:**
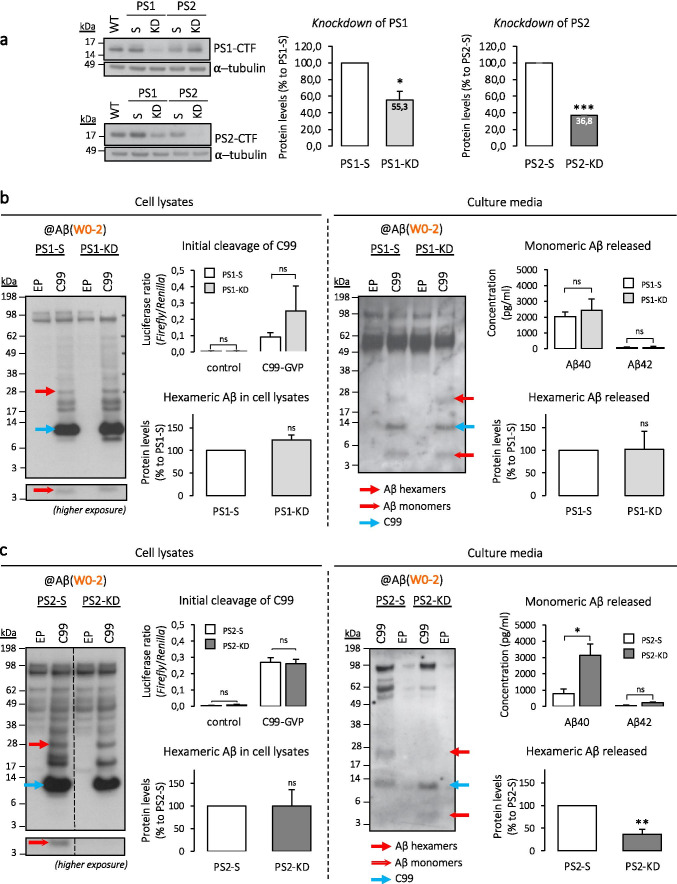
Contribution of presenilins to the production of hexameric Aβ assemblies. **a** SH-SY5Y *knockdown* (KD) cell lines were generated using CRISPR-Cas9, with guide RNA vectors targeting either human *PSEN1* (PS1-KD) or *PSEN2* (PS2-KD) genes. Control cells were transfected with respective scrambled sequences. Left, a representative Western blot; middle and right, quantitative decrease in PS1 and PS2 protein levels in KD compared to S cells (for all conditions, see Supplementary Fig.S2). *N* = 3. One-sample *t* test with hypothetical value set as 100: *p < 0.05, ***p < 0.001 (S vs KD, in PS1: *p* = 0.03; in PS2: *p* = 0.0001). *WT* wild-type, *S* scrambled. **b**, **c** Initial cleavage ability was monitored by a reporter gene assay. The release of APP intracellular domain (AICD) from a tagged C99-GVP substrate was measured by the Gal4-*Firefly* reporter gene. Results are represented as *Firefly*/*Renilla* luciferases ratios, with *Renilla* serving as a transfection-efficiency control. The profile of Aβ production was assessed after transfection with either an empty plasmid (EP) or C99, using Western blotting and ECLIA immunoassay, in PS1-KD vs PS1-S (in **b**) and in PS2-KD vs PS2-S (in **c**). Media samples were lyophilized prior to analysis. Dashed lines indicate that proteins were run on the same gel, but lanes are not contiguous. Luciferase assays (initial cleavage of C99): *N* = 4 each. One-way ANOVA with Tukey's multiple comparison test: ns = non-significant (S vs KD, in PS1 control: *p* > 0.99; in PS1 C99-GVP: *p* = 0.10; in PS2 control: *p* = 0.99; in PS2 C99-GVP: *p* = 0.99). Western blots quantitative analyses (hexameric Aβ, in cell lysates and released, relative to C99 and expressed as a % to S): *N* = 3 each. One-sample *t* test with hypothetical value set as 1: *ns* non-significant, **p < 0.01 (S vs KD, in PS1 cell lysates: *p* = 0.16; in PS1 media: *p* = 0.97; in PS2 cell lysates: *p* = 0.99; in PS2 media: *p* = 0.01). ECLIA assays (monomeric Aβ released): *N* = 5 each. Mann–Whitney test: *ns* non-significant, *p < 0.05 (S vs KD, in PS1 Aβ_40_: *p* > 0.99; in PS1 Aβ_42_: *p* > 0.99; in PS2 Aβ_40_: *p* = 0.04; in PS2 Aβ_42_: *p* = 0.12)

For this, we evaluated the profile of Aβ species formed in human cells expressing the C99 fragment and lacking either PS1 or PS2. We used a CRISPR-Cas9 editing approach [34, 35] to generate PS1 and PS2 *knockdown* (KD) neuron-derived human cell lines (SH-SY5Y cells). SH-SY5Y cells readily produced hexameric-like Aβ assemblies in our conditions (Fig. 2c). Cells were stably transfected with a CRISPR-Cas9 expression system targeting either *PSEN1* or *PSEN2* genes, and selected using fluorescent (FACS) and antibiotic resistance double-selections. Scrambled (S) target sequences for both the *PSEN1* and *PSEN2* genes were used for the generation of control cell lines. After subcloning, the expression of both presenilins (PSs) was verified by Western blotting (Fig. 3a) which showed a 44.7% and 63.2% reduction of PS1 and PS2 protein levels, respectively. To note, the levels of the other—non-targeted—PS was not significantly affected (Supplementary Fig.S3).

We first assessed the ability of the KD cells to perform the initial cleavage of the C99 substrate, releasing the APP intracellular domain (AICD). The AICD release from a tagged C99-GVP substrate was measured by a Gal4 reporter gene assay, as described previously [21, 32]. Results revealed an efficient cleavage of the C99-GVP construct in both the PS1-KD and PS2-KD cells, when compared to PS1-S and PS2-S controls, respectively. This suggests that neither the PS1 nor PS2 *knockdown* blocked the processing at the initial γ-secretase cleavage site. We next investigated the profile of Aβ production in these cell lines by combining Western blotting and ECLIA techniques. Results indicated that the reduction in PS1 levels had no significant effect on the profile of Aβ present inside or outside of the cell (Fig. 3b), with no significant decrease in monomeric Aβ measured in culture media. This was quite an unexpected observation that could be explained by the fact that only around 50% of PS1 *knockdown* was achieved in our model. The remaining PS1-dependent γ-secretase activity could be sufficient to efficiently process APP-derived substrates. However, while the formation of intracellular hexameric Aβ was similar between PS2-KD and corresponding control cells, intracellular monomeric Aβ was no longer detectable when PS2 expression was reduced. Concomitantly, the extracellular Aβ assembly profile was altered in PS2-KD cells, with an increase in monomeric form and an acute decrease in hexameric Aβ, suggesting that the extracellular release of the hexameric-like Aβ assemblies is dependent on the presence of PS2 (Fig. 3c). This would illustrate that the absence of PS2 (i) favors the accumulation of monomeric extracellular Aβ and (ii) leads to decreased intracellular monomeric Aβ and decreased extracellular aggregates. In other words, PS2-dependent γ-secretases could generate aggregation-prone intracellular Aβ that is eventually released as an aggregate in the extracellular space.

**Fig. 4:**
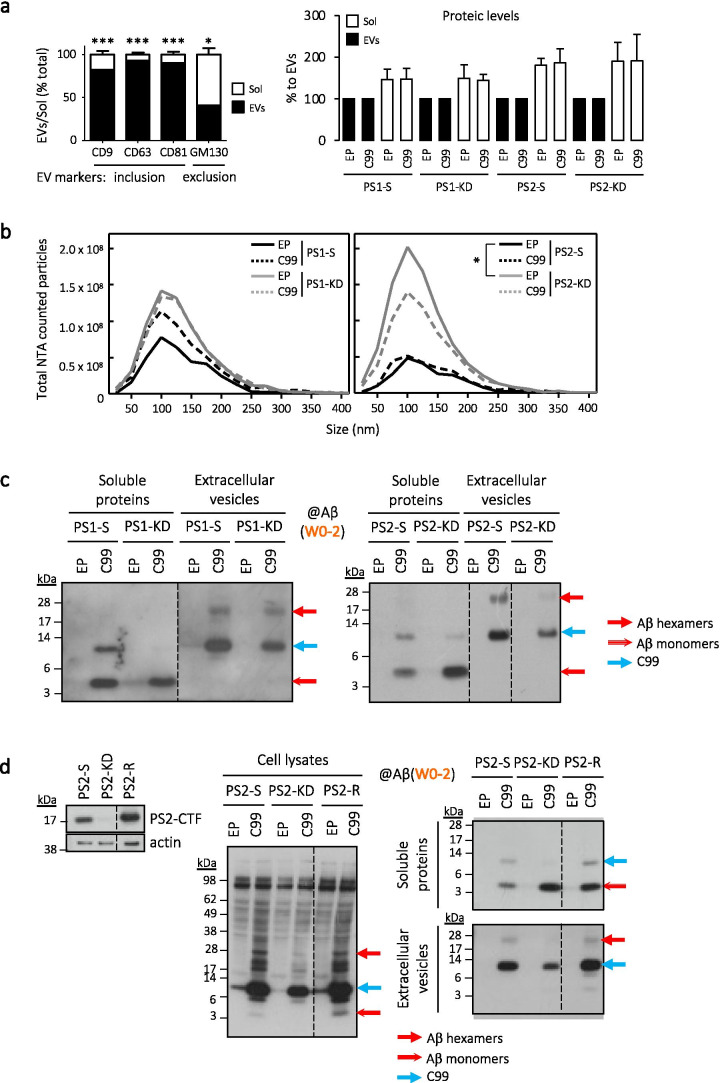
PS2-dependent release of hexameric Aβ assemblies in extracellular vesicles (EVs). Complete media of PS1-S, PS1-KD, PS2-S, and PS2-KD cells underwent an ultracentrifugation process to separate putative enrichment of EVs, in pellet, from soluble proteins. **a** The efficiency of EVs isolation was confirmed by plate-based Europium-immunoassay (left panel) showing an enrichment of EVs inclusion markers CD9, CD63, and CD81, while EVs exclusion marker GM130 was lower in EVs as compared to soluble fractions. *N* = 6. Mann–Whitney test: *p < 0.05, ***p < 0.001 (CD9 (*n* = 22), CD63 (*n* = 22), and CD81 (*n* = 18): *p* < 0.0001; GM130 (*n* = 19): *p* = 0.0362). Quantification of protein levels by BCA (right panel) showed larger protein amounts in soluble than EVs fractions, ruling out the specific enrichment of inclusion markers due to higher content in proteins. **b** EVs ultracentrifugation pellets were counted for number of particles of 70-400 nm by nanoparticle tracking analysis (NTA). *n* = 9 in *N* = 3 independent experiments. Two-way ANOVA with Bonferroni’s multiple comparison: *p < 0.05 (PS2-KD EP vs PS2-S EP: *p* < 0.05). **c** Both EVs and soluble extracts were monitored by Western blotting with the W0-2 antibody. Dashed lines indicate that proteins were run on the same gel, but lanes are not contiguous. **d** Human PS2 was re-expressed in PS2-KD cells to assess the restoration of observed phenotypes. The efficiency of re-expression was monitored using the PS2-CTF antibody in PS2 rescued (R) cells with comparison to PS2-S and PS2-KD cells (left panel). Cell lysates (middle panel) as well as extracellular soluble proteins (upper right panel) and EVs (lower right panel) were monitored by Western blotting with the anti-Aβ (W0-2) antibody. *EP* empty plasmid, *EV(s)* extracellular vesicle(s), *Sol* soluble proteins fraction

To note, PS2 [23, 41], as well as APP and intermediate fragments of its metabolism [42, 43], were previously found in endo-lysosomal compartments and extracellular vesicles (EVs). We examined whether the extracellular hexameric-like Aβ assemblies were present in EVs. A specific ultracentrifugation procedure was performed to separate EVs from soluble proteins in the media of PS1-KD, PS2-KD, and respective control cells (PS1-S, PS2-S). The accuracy of the EVs separation was validated by the Europium-immunoassay with, in the EVs, significantly increased levels of inclusion markers CD9, CD63, and CD81 and lower content of the exclusion marker GM130 (Fig. 4a, left panel). The specific enrichment of inclusion markers due to a higher protein content in EVs was ruled out since whole-protein assay showed larger protein amounts in soluble than EVs fractions (Fig. 4a, right panel). Importantly, extracellular monomeric Aβ was found exclusively in the soluble proteins fraction while hexameric Aβ was confined exclusively in vesicles (Fig. 4c), in agreement with the recent observations on “Aβ-like” oligomeric species [44]. To note, EVs size distribution was similar between all conditions but the number of EVs was higher in PS1-KD and PS2-KD as compared to respective controls (Fig. 4b), indicating that the decrease in hexameric-like Aβ assemblies observed in PS2-KD is not due to a decrease in EVs formation.

To assess whether the observed phenotype was truly dependent on PS2, we have re-expressed human wild-type PS2 in SH-SY5Y PS2-KD cells by lentiviral infection followed by selection. This resulted in efficient expression of PS2, as can be observed in Fig. 4d with a strong signal for PS2-CTF in PS2 rescued cells (PS2-R). We have repeated the Western blotting analysis on lysates and media samples (processed by ultracentrifugation to differentiate soluble proteins and EVs) from these cells, in parallel to PS2-S and PS2-KD cells. We found that, upon PS2 re-expression (PS2-R), the intracellular pool of Aβ monomers and the extracellular release of Aβ hexamers were both restored (Fig. 4d).

Together, our results indicate that PS2 plays a critical role in the release of hexameric-like Aβ assemblies, liberated in the extracellular milieu through EVs. Importantly, proteins confined in EVs were previously suggested to play a pivotal role in amyloid plaques formation and AD [45], suggesting that the Aβ hexamers identified here might be able to trigger specific aspects of the amyloid pathology. We thus next sought to investigate if similar hexameric-like Aβ assemblies were present in a pathological context.

### Hexameric-Like Aβ Assemblies in the Context of Amyloid Pathology

**Fig. 5:**
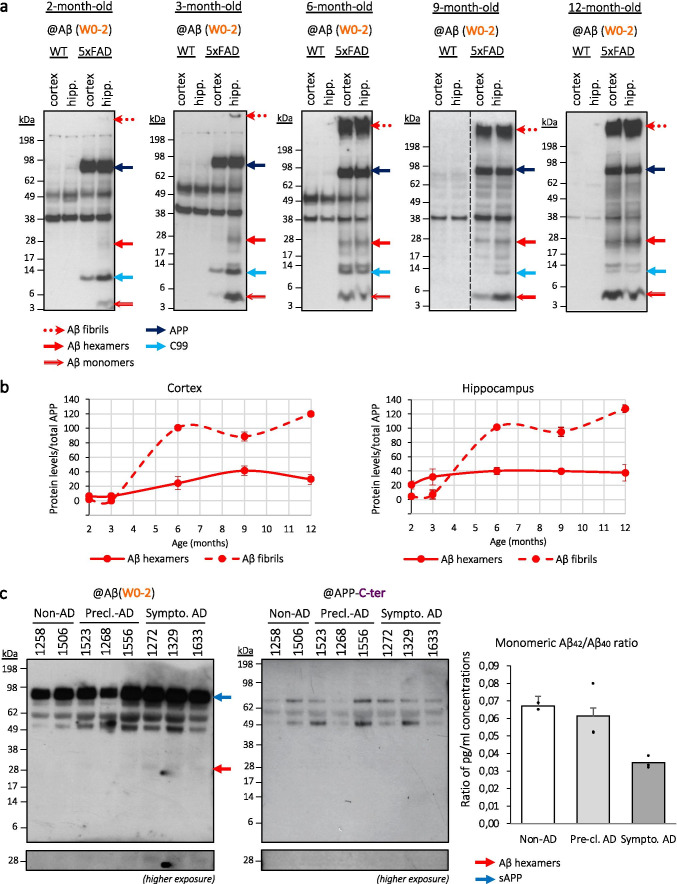
Identification of hexameric-like Aβ assemblies in the context of AD. **a** Detection of Aβ assemblies in brain samples from an amyloid mouse model (5xFAD). Cortices and hippocampi of euthanized mice were lysed and analyzed by Western blotting with the anti-Aβ (W0-2) antibody. Aβ fibrils appear stuck in the wells and hexameric-like Aβ assemblies are detected at ~ 28 kDa. To note, C99 fragments (~ 10 kDa) and Aβ monomers (~ 4.5 kDa) are also detected in all 5xFAD samples and reflect an efficient metabolism of the human APP protein expressed in these mice. Dashed lines indicate that proteins were run on the same gel, but lanes are not contiguous. *Hipp.* hippocampus. **b** The signal intensities of Aβ hexamers and Aβ fibrils were quantified relatively to the APP signal. Samples used for quantitative analysis derived from the same experiment, with Western blots processed in parallel. The displayed graphs represent the profile of Aβ assemblies as related to both the analyzed brain area (cortex, hippocampus) and the age (2, 3, 6, 9, 12 months of age) (min *N* = 3 each). **c** Identification of ~ 28 kDa, hexameric-like, Aβ assemblies in the cerebrospinal fluid (CSF) of AD patients. Western blotting analysis was performed using the anti-Aβ (W0-2) and anti-APP-C-ter antibodies. Higher exposures were performed for a better appreciation of the bands of interest; full images can be found in Supplementary Fig.S5. Dosage of monomeric Aβ_42_/Aβ_40_ by ECLIA immunoassay confirmed the correct classification of individuals, with a reduction in ratio along with AD progression. *sAPP* soluble APP, *Pre-cl.* Pre-clinical, *Sympto.* symptomatic

We investigated the presence of similar Aβ assemblies in mice expressing familial AD (FAD) mutations. We readily detected ~ 28 kDa Aβ assemblies (Fig. 5a) in brain extracts of 5xFAD mice [29], likely corresponding to those we have identified as hexameric Aβ in a cellular model. Interestingly, the intensity of hexameric-like Aβ detection in 5xFAD mice brains increased with age. To note, the detection of these assemblies preceded that of high molecular weight Aβ assemblies (> 198 kDa) likely corresponding to fibrils, which are recognized as the major indicator of the development of amyloid deposits in the 5xFAD model [29, 46]. Quantitative analysis of Aβ hexamers (~ 28 kDa) and fibrils (> 198 kDa) relative to the human APP expressed in mice brains confirmed the appearance of the ~ 28 kDa Aβ assemblies as an early event (Fig. 5b). These assemblies accumulated first in the hippocampus of the mice, as early as 2-month-old, while their detection in cortical regions peaked at 3 to 6 months of age. This is in line with the staging of amyloid pathology observed in human AD and suggests that hexameric-like Aβ assemblies might serve as an early indicator of amyloid pathology development and as an important contributor to its progression.

In addition, cerebrospinal fluid (CSF) samples from cognitively affected patients (diagnosed with non-AD dementia, pre-clinical AD or symptomatic AD) were monitored with the same Western blotting approach (Fig. 5c, left panel). Only AD-related patients revealed the presence of ~ 28 kDa Aβ assemblies. Although the amounts of assemblies detected in the CSF remain quite low, their signal increases in symptomatic AD cases with respect to control or pre-clinical AD. This evidences the presence of hexameric-like Aβ assemblies in the context of AD pathology for the first time. Full images of higher exposures can be found in Supplementary Fig.S5, while more detailed information on neurological examination and PET analyses conducted on the patients are displayed in Supplementary Table.S1. The same CSF samples were used for a quantitative analysis of monomeric Aβ isoforms by ECLIA immunoassay and revealed an overall reduction in the Aβ_42_/Aβ_40_ ratio in AD patients (Fig. 5c, right panel). This reduction correlated with the severity of AD symptoms shown by the patients (see raw values in Table.1), in agreement with previous reports [47]. On the contrary, relative quantification of hexameric-like Aβ levels detected by Western blotting, using soluble APP (sAPP) as an intrasubject control, revealed an increase in the levels of hexameric-like Aβ assemblies alongside the progression of AD (Table 1). This suggests a direct correlation between the reduced proportion of monomeric Aβ_42_ and its aggregation in the hexameric-like ~ 28 kDa assemblies, in line with the previous evidences of an inverse correlation between monomeric Aβ and higher Aβ assemblies in AD [48, 49]. In our study, statistical analysis revealed that 48.8% of the Aβ_42_/Aβ_40_ ratio variance can be explained by the increase in hexameric Aβ formation.

**Table 1:** Inverse correlation between monomeric and hexameric-like Aβ in human CSF samples

Inclusion *n*°	Classification	Aβ_42_/Aβ_40_	Hexameric Aβ/sAPP
1258	Non-AD	0.069	0.079
1506		0.065	0.055
1523	Pre-clinical AD	0.052	0.075
1268		0.080	0.084
1556		0.052	0.092
1272	Symptomatic AD	0.033	0.140
1329		0.039	0.224
1633		0.032	0.143

CSF samples from eight subjects were monitored in this study: patients *n°* 1258 and 1506 did not exert any AD-related features. Patients *n°* 1523, 1268, and 1556 were diagnosed with pre-clinical AD and patients *n°* 1272, 1329, and 1633 with symptomatic AD. Ratios of monomeric Aβ_42_ over monomeric Aβ_40_, and of hexameric-like Aβ over soluble APP (sAPP) were obtained from ECLIA and Western blot quantifications, respectively (see Fig. 5). Pearson’s *R* correlation test: *R* =  − 0.70 (*R*-squared = 0,49), *p* = 0.05

### Cell-Derived Hexameric Aβ Aggravates Amyloid Deposition in an AD Mouse Model and Decreases Cell Viability in Primary Neurons Expressing Human APP

**Fig. 6:**
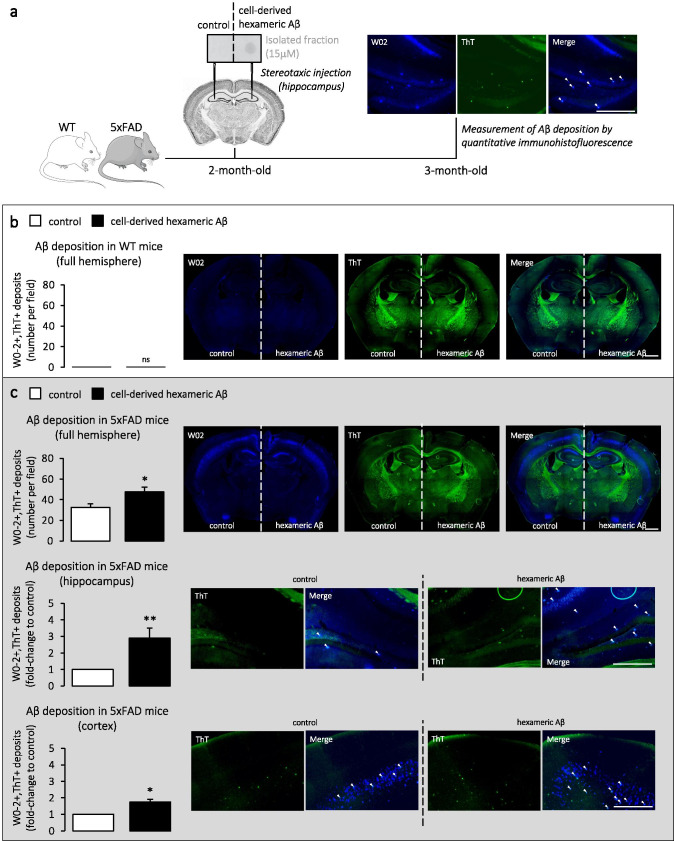
Intracerebral injection of cell-derived hexameric Aβ in WT and 5xFAD mice. **a** Experimental workflow. 2-month-old mice were deeply anesthetized, placed in a stereotaxic apparatus and bilaterally injected with 2 μl of either 15 μM GELFrEE™-isolated Aβ hexamers (C42) or control (EP) in the hippocampus (A/P − 1.94; L +  − 2.17; D/V − 1.96; mm relative to bregma). Both fractions were analyzed by dot blotting prior to injection. 30 days later, mice were transcardially perfused, brains were fixed, and coronally sectioned (50 μm). Immunostaining was performed on free-floating sections using the anti-human Aβ (W0-2) antibody as a marker for Aβ and the Thioflavin T (ThT) dye as a marker for fibrillar deposits. W0-2 and ThT staining were detected with FITC/Cy5 and GFP filters, respectively. Right panel displays an example of double-positive counting. Scale bar = 400 μm. **b**, **c** Quantification of fibrillar deposits in full hemispheres of WT (in **b**) and 5xFAD brains (in **c.** upper panel) injected with control vs hexameric Aβ. Scale bar = 1000 μm. *n* = 32 slides from *N* = 8 mice for both WT and 5xFAD. Mann–Whitney test: *ns* non-significant, *p < 0.05 (in WT: *p* > 0.99 control vs hexameric Aβ; in 5xFAD: *p* = 0.04 control vs hexameric Aβ). For transgenic mice, deposits were also classified according to the two most affected brain regions, hippocampus and cortex, as a function of the control-injected hemisphere (in **c** middle and lower panel, scale bar = 400 μm). A 2.90-fold and a 1.74-fold increase were observed in the hippocampus and cortex, respectively. One-sample Wilcoxon signed-rank test with hypothetical value set at 1: *p < 0.05, **p < 0.01 (in 5xFAD hippocampus: *p* = 0.008 control vs hexameric Aβ; in 5xFAD cortex: *p* = 0.02 control vs hexameric Aβ)

To further assess the potential of cell-derived hexameric Aβ assemblies to trigger amyloid pathology, we performed hippocampal stereotaxic injections in two mouse models: (i) WT mice (C57BL/6) to assess the potential of Aβ hexamers to form amyloid deposits in a previously amyloid-free context, and (ii) mice developing amyloid pathology (5xFAD) to mimic a situation where the hexamers are incubated with pre-existing Aβ, to serve as seeds driving the assembly and deposition of Aβ produced in the brain. Experimental workflow is represented in Fig. 6a. The isolated assemblies were obtained by W0-2-immunoprecipitation and GELFrEE™ separation of C42-expressing CHO cells media as described above (Fig. 1) [15]. The corresponding GELFrEE™ fraction of cells expressing the empty plasmid (EP) was used as a control. The fraction of cell-derived hexameric Aβ was diluted from 150 to 15 μM prior to intracerebral injection. The control fraction was diluted in a similar manner. Specific detection of diluted cell-derived hexameric Aβ was confirmed by dot blotting with the W0-2 antibody (Fig. 6a, left panel). 2-month-old WT or 5xFAD mice were injected in the hippocampus of the left and right hemisphere with 2 μl of EP (control) and C42 (cell-derived hexameric Aβ) diluted fractions, respectively. To evaluate Aβ deposition, mice were sacrificed 30 days after stereotaxic injection and coronal sections of fixed brains were co-stained with the human-specific W0-2 antibody for Aβ and the Thioflavin T (ThT) dye for fibrillar aggregates. Quantitative analysis of Aβ deposition was performed by counting double-positive dots (as indicated in Fig. 6a). The results showed that cell-derived hexameric Aβ assemblies do not have the ability to form fibrillar deposits by themselves in a WT brain (Fig. 6b), but are capable of enhancing the deposition of Aβ already present in the 5xFAD brain (Fig. 6c). In transgenic mice, the overall deposition of Aβ in the hemisphere injected with cell-derived hexameric Aβ showed a significant 1.47-fold increase when compared to the control-injected hemisphere (average (± SEM) of 32.39 (± 3.49) and 47.50 (± 4.74) deposits per field in control- and hexamer-injected hemispheres, respectively). Deposits were further investigated in the two regions mainly affected by amyloid pathology in AD: the hippocampus and the cortical areas (Fig. 6c). As expected, the highest increase in Aβ deposition was observed in the hippocampal region, where stereotaxic injections were performed (2.90-fold increase relative to control). However, levels of Aβ deposits were also significantly increased by a 1.74-fold in the cortex. This suggests that the injected cell-derived hexameric Aβ assemblies are able to propagate from the hippocampal formation to associated cortical regions to promote amyloidosis.

**Fig. 7:**
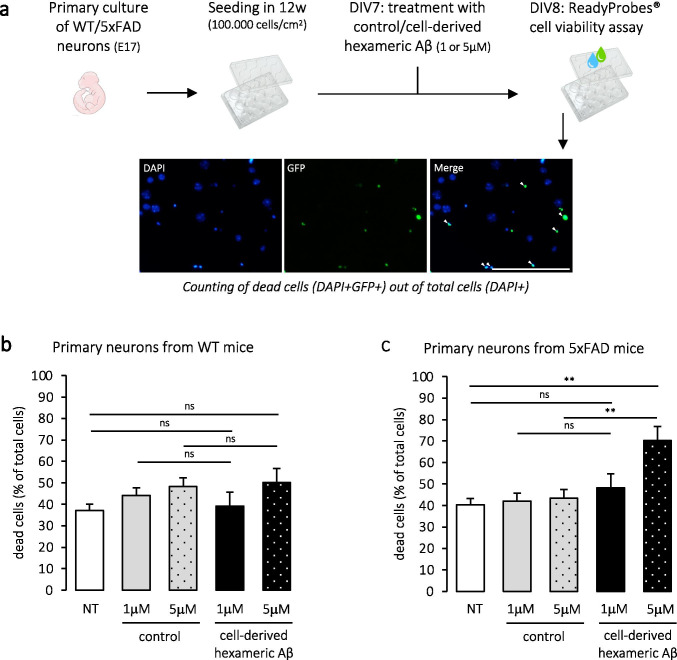
Cell-derived hexameric Aβ assemblies are only cytotoxic in primary neurons that express amyloid proteins. **a** Experimental workflow. Primary neurons were isolated from wild-type (WT) or transgenic (5xFAD) mouse embryos at stage E17 and cultured for 8 days in vitro (DIV). At DIV7, cells were incubated for 24 h with 1 µM or 5 µM of cell-derived hexameric Aβ or control, isolated from the media of C42- and EP-expressing CHO cells, respectively. Cell viability was assessed using the ReadyProbes® assay and fluorescent staining was captured at an EVOS® FL Autofluorescence microscope. A representative image of the assay is shown. Scale bar = 50 μm. **b**, **c** Quantification of the proportion of dead cells compared to the total cells in WT (in **b**) and 5xFAD cultures (in **c**). Total number of cells counted (number of dead cells counted in brackets) was as follows in WT: *n* = 1183(439), 1070(472), 650(314), 813(318), 797(400), and 5xFAD: *n* = 528(212), 640(270), 1019(442), 465(224), 775(544) for NT, control (equivalent of 1 μM), control (equivalent of 5 μM), hexameric Aβ (1 μM), and hexameric Aβ (5 μM) respectively. *NT* not treated. *N* = 4 independent experiments in WT, *N* = 3 independent experiments in 5xFAD. One-way ANOVA with Tukey's multiple comparison test: *ns* non-significant, *p < 0.05, **p < 0.01 (in WT: *p* = 0.99 NT vs hexameric Aβ (1 μM), *p* = 0.38 NT vs hexameric Aβ (5 μM), *p* = 0.95 control (1 μM) vs hexameric Aβ (1 μM), *p* = 0.97 control (5 μM) vs hexameric Aβ (5 μM); in 5xFAD: *p* = 0.70 NT vs hexameric Aβ (1 μM), *p* = 0.004 NT vs hexameric Aβ (5 μM), *p* = 0.85 control (1 μM) vs hexameric Aβ (1 μM), and *p* = 0.009 control (5 μM) vs hexameric Aβ (5 μM))

We finally assessed whether isolated cell-derived hexameric Aβ was capable of exerting neurotoxic effects. To that end, we cultured primary neurons from wild-type (WT) and transgenic (5xFAD) embryos and treated them after 7 days of differentiation in vitro (DIV) with isolated hexameric Aβ assemblies. As for in vivo seeding assays, Aβ hexamers were obtained by W0-2-immunoprecipitation and GELFrEE™ separation of C42-expressing CHO cells media and the corresponding fraction of EP-expressing CHO cells media was used as a control. Two final concentrations were tested; 1 μM and 5 μM. 24 h after treatment, cell viability was assessed using a ReadyProbes® assay and a percentage of dead cells out of the total cells was quantified (Fig. 7a). Results showed no significant cytotoxic effect at tested concentrations on primary neurons cultured from WT mice (Fig. 7b), even though both are above the reported neurotoxic concentrations from preparations of synthetic oligomeric Aβ [6, 50]. This suggests that the identified assemblies are not cytotoxic per se, at least in these experimental conditions. However, primary neurons cultured from 5xFAD mice, which can serve as an amyloid model in vitro [51–53], displayed increased cell death when exposed to 5 μM of cell-derived hexameric Aβ (Fig. 7c). Importantly, this indicates that hexameric Aβ assemblies may have the ability to cause toxic effects only when there is pre-existing Aβ in the neuronal environment. This therefore implies that such cytotoxic properties require the intermediate seeding of other available Aβ forms.

## Discussion

The identification of fibrillar Aβ as the main component of amyloid plaques present in AD has led to the extensive investigation of the Aβ self-assembly process and, as a result, to the identification of many intermediate oligomeric assemblies of Aβ. Today, it is widely admitted that such non-fibrillar, soluble assemblies can exert a widespread neurotoxic effect and should be the main target of therapeutic prevention or intervention. Among Aβ oligomers, hexameric Aβ has repeatedly been identified as a key assembly in vitro [3, 8, 11, 54]. Studies performed on synthetic preparations of Aβ have shed light on the crucial role of Aβ hexamers in the nucleating step of Aβ self-assembly [11–13, 55]. However, such studies have not, to the best of our knowledge, characterized the production of hexameric Aβ in a cellular environment, nor assessed its toxic potential and relevance to AD pathogenesis. Our work shows that specific ~ 28 kDa Aβ assemblies are produced in a wide range of biological models. Using CHO cells, we were able to confirm the nature of these assemblies as hexameric Aβ_42_ [15]. The question of whether these assemblies are exclusively formed by six Aβ_42_ monomers associated together would require extensive analytical biochemistry investigation, which are of real interest, but beyond the scope of this study. Nevertheless, a major finding here is that the Aβ assemblies we investigated across different cell lines (i) have an apparent molecular weight around 28 kDa; (ii) are recognized by W0-2, 6E10, and anti-Aβ_42_ antibodies but not by anti-APP-C-ter or anti-Aβ_40_ antibodies; and (iii) are affected by the *knockdown* of PS2, a catalytic subunit of the γ-secretase complex. Our recently reported aggregation assays revealed a seeding potential of the isolated assemblies when incubated with recombinant monomeric Aβ in vitro [15]. Together, this clearly indicates that the identified Aβ assemblies contain Aβ_42_ and have not only the theoretical size, but also the expected properties of Aβ hexamers.

With this important observation in mind, we sought to better understand the cellular context in which hexameric Aβ is produced. In particular, we assessed the respective involvement of both presenilins in its production. Indeed, the distinct subcellular localizations [23] and differential substrate specificities [21] of PS1 and PS2 control the production of different Aβ pools. Production of Aβ can differ considerably between cellular compartments [23]. Pathological Aβ formation is related to dysfunction of the endocytic pathway, and PS1 and PS2 are differentially distributed between the secretory compartments and the late endosomes/lysosomes, to which PS2 is shuttled [23]. Our results showed the absence of any significant change in the processing of C99 or in the release of Aβ when cells have a nearly 50% reduction of PS1 protein levels. This was rather unexpected regarding the previous reports on the preponderant importance of PS1 for γ-secretase substrates cleavage and overall Aβ production [17–21]. One can imagine, as already mentioned above, that the reduction of PS1 protein levels is not significant enough to observe any effect. PS1 *knockdown* might induce compensatory mechanisms and still ensure its primary function even when its protein levels are reduced by half. In the PS2 *knockdown* cells, the reduction of PS2 protein levels by just over 60% was sufficient to cause clear changes in Aβ production. While the initial cleavage of the C99 construct remained unaffected, the production of monomeric Aβ was strongly diminished inside the cell. This is linked with the previous reports suggesting that the intracellular pool of Aβ is mostly generated through the activity of PS2 [23]. In an opposite manner, the production of monomeric Aβ was strongly increased in the extracellular medium. In particular, the Aβ_40_ isoform was increased in the medium of PS2-KD cells. The levels of the ~ 28 kDa hexameric Aβ assemblies were unchanged in the cell lysates but strongly diminished in the extracellular environment.

Importantly, we observed for the first time that specific Aβ assemblies were enriched in extracellular vesicles, while monomeric Aβ was present solely in the soluble fraction. Although the amount of EVs tended to increase upon *knockdown* of PS1/PS2, it did not seem to impact Aβ fate. As PS1 and PS2 have been, respectively, shown to produce the extra- and intracellular pools of Aβ [23], the direct release of soluble Aβ outside the cellular environment is likely to rely mostly on the action of PS1, while the intracellular pool is likely to mainly result from PS2-dependent γ-secretase activity in endocytic compartments. Previous reports suggest that the PS2-dependent cleavage mainly occurs in a specific subset of endosomes and leads to the presence of Aβ in multivesicular bodies (MVBs) which, after fusion with the cell membrane, can release their Aβ-containing intraluminal vesicles as EVs in the extracellular milieu [45].

As PS2-dependent cleavage was reported to predominantly produce intracellular aggregation-prone Aβ_42_ [23], it is likely that the monomeric Aβ formed along the endocytic-EVs pathway corresponds to Aβ_42_ and extensively aggregates inside vesicles, evidently as hexameric Aβ_42_. On the other hand, the soluble monomeric Aβ that is produced through the PS1-dependent secretory pathway likely corresponds to Aβ_40_, which might not be able to aggregate. This is supported by observations reported in our recent biochemical in vitro study [15], indicating that soluble monomeric Aβ present in the medium of C99-expressing CHO cells corresponds to non-aggregating Aβ_40_. To note, the increase in the extracellular levels of soluble monomeric Aβ_40_ observed in PS2-KD cells likely reveals a PS1-dependent compensatory mechanism taking place upon PS2 *knockdown*.

Together, our results in both *knockdown* cell lines drive towards the hypothesis that extracellular hexameric Aβ found in EVs might stem from MVBs and thus from the intracellular pool of Aβ, a process controlled by PS2 activity. The identification of such a specific role for PS2 in the release of hexameric Aβ is of particular importance. It is quite promising in the hope of re-evaluating Aβ modulators and developing therapeutic agents specifically targeting PS2-dependent γ-secretase complexes. To note, PS1 and PS2 were reported to have different sensitivities to γ-secretase inhibitors [21]. Importantly, the intracellular pool of Aβ, generated by PS2, has been repeatedly associated with the progression of AD [56–59]. FAD mutations in *PSEN2* have been shown to dramatically increase the proportion of longer Aβ fragments intracellularly, accelerating its assembly. Further, a subset of familial mutations on *PSEN1* have been reported to shift the localization of the PS1 protein to fit that of PS2 [23]. Thus, it is likely that the PS2-dependent production of aggregation-prone Aβ inside intracellular compartments and its resulting accumulation and excretion are enhanced in the context of AD.

In line with this, we also report for the first time the presence of hexameric-like Aβ assemblies in brain extracts from a well-characterized amyloid mouse model (5xFAD) as well as in the CSF of AD patients. This supports that hexameric Aβ may act as an important actor in the amyloid pathology. In the 5xFAD model, the presence of hexameric-like Aβ assemblies followed a regional pattern of progression that corresponds to the neuropathological, clinical staging of human AD pathogenesis [60]. Indeed, the ~ 28 kDa assemblies were detected first in the hippocampus, as early as 2-month-old, and further spread to the cortex starting from 3- to 6-month-old. On the contrary, amyloid plaques formation in the 5xFAD mice have previously been reported to appear first in deep layers of the cortex and in the subiculum, and to later spread to the hippocampus as mice aged [29]. As the spreading properties of soluble Aβ oligomers are likely to depend on the initial site where they are formed, investigating the aggravation of amyloid pathology upon injection of cell-derived hexameric Aβ in the cortical areas might bring important evidence to support this hypothesis. Meanwhile, the pattern of detection of hexameric-like Aβ assemblies reported here suggest that they could represent early biomarkers of amyloid pathology. The detection of similar ~ 28 kDa assemblies in CSF extracted from human patients diagnosed with pre-clinical AD and symptomatic AD supports this hypothesis.

The isolation of hexameric Aβ from our CHO cell model has also allowed for the characterization of its pathological properties. More precisely, we assessed the contribution of isolated cell-derived hexameric Aβ to both (i) amyloid deposition upon stereotaxic injection in mouse brains and (ii) cytotoxicity upon direct exposure to primary neurons. We assessed this in both (i) wild-type (WT) mice, to evaluate the intrinsic pathological properties of hexameric Aβ, and (ii) transgenic 5xFAD mice, to evaluate the ability of hexameric Aβ to aggravate the development of amyloid pathology.

Using WT mice, we found that hexameric Aβ assemblies were not able to induce Aβ deposition nor elicit any cytotoxic effect per se. To note, Aβ deposition in vivo was assessed in a timeframe of 30 days and one cannot exclude that pathogenic events might take place upon longer incubation time. In addition, the absence of Aβ deposits formation (stereotaxic injections) or cytotoxicity (cultured neurons) induced by cell-derived hexameric Aβ does not exclude its ability to alter brain function apart from amyloid deposition or cytotoxicity. Indeed, cellular dysfunctions or alterations of neuronal connectivity might occur in our WT models without leading to detectable amyloidosis or significant neuronal death. The absence of Aβ deposits after injection of cell-derived hexameric Aβ in the brain of WT mice also does not exclude the possibility that the stable seeds injected might not directly cause amyloidosis in the injected animals, but persist in the brain and retain pathogenic properties, as was previously shown with second-transmission studies [7]. Interestingly, observations taken from aggregation assays revealed that hexameric Aβ assemblies are very stable when incubated alone in vitro, and unable to further aggregate into higher molecular weight complexes [15]. The lack of direct neurotoxic effects of the studied assemblies is thus in agreement with the fact that (i) the process of Aβ self-assembly is thought to be vital in mediating cytotoxicity [33, 61] and that (ii) the pathological properties of Aβ oligomers could rely not only on their synaptotoxic effects but also on their seeding properties, propagating amyloid pathology throughout the brain parenchyma.

Considering the suggested role of hexameric Aβ as a nucleus for Aβ self-assembly, we wondered whether the harmful potential of Aβ hexamers could be unraveled when pre-existing Aβ species are present. Indeed, a major observation in this study is the ability of cell-derived hexameric Aβ to act as a seeding nucleus and cause both aggravation of cerebral Aβ deposition and cytotoxicity in primary neurons of transgenic 5xFAD mice. These mice express five familial AD mutations that together trigger Aβ_42_ overproduction and result in a rapid and severe development of amyloid pathology [29, 46]. 5xFAD mice therefore represented an accurate model to assess the nucleating properties of cell-derived hexameric Aβ assemblies in vivo in a reasonable timeframe. An earlier onset of Aβ aggregation in this model was previously reported upon single intracerebral injection of brain homogenates containing oligomeric Aβ, following a prion-like seeding mechanism [62]. Aβ oligomers have been further suggested to be early triggers of the seeding process, as their depletion by passive immunization delays Aβ aggregation and leads to a transient reduction of seed-induced Aβ deposition [63]. Our study has the advantage of focusing on a specific cell-derived Aβ aggregate, that is heavily involved in the processes of nucleation and seeding as shown in recent in vitro studies [11, 15]. We performed intracerebral injections at 2 months of age, when amyloid deposition begins in the 5xFAD mice [29]. The significant increase of Aβ deposition observed in hexamer-injected hemispheres suggests that hexameric Aβ is indeed able to promote amyloidosis. As very recent in vitro studies from our group revealed the ability of the isolated Aβ hexamers to drive the aggregation of synthetic monomers of Aβ in vitro [15], it is likely that the enhancement in Aβ deposition observed here in vivo relies on the same process of nucleation, with Aβ hexamers serving as a template for aggregation. The greater increase of Aβ deposition in the hippocampus when compared to the cortex of the injected mice supports this hypothesis, as hexameric Aβ is likely to seed and promote Aβ aggregation to a higher rate at the injection site. Still, the significant aggravation of Aβ deposition observed in the cortex also suggests that hexameric Aβ is able to spread throughout the brain, as rapidly as in 30 days. Together, these observations suggest that hexameric Aβ is a key factor in the amyloidosis process, with seeding properties and ability to spread through connected brain regions, and might serve when naturally present as an early indicator of Aβ deposition. Cytotoxic assays on primary neurons derived from transgenic 5xFAD mice were also performed, since cultured neurons from AD transgenic animal models express APP metabolites involved in amyloid pathology and can reflect AD phenotypes in vitro [51–53]. We observed a significant increase in the proportion of cell death when neurons were treated with 5 μM of cell-derived hexameric Aβ. This suggests that these specific assemblies can indeed exert a toxic effect in a FAD context, where human Aβ to seed is available.

## Conclusions

Altogether, our findings have shed light on a particular cell-derived Aβ assembly that corresponds to an Aβ_42_ hexamer. An insight in cellular mechanisms at stake suggests a strong contribution of PS2 to the formation of this particular Aβ oligomer, which is released in the extracellular milieu inside vesicles. This in line with the previous reports linking the restricted location of PS2 in acidic compartments of the endocytic pathway, from which extracellular vesicles can form, to the production of more aggregation-prone Aβ. Combining in vitro and in vivo approaches, we have revealed an absence of detrimental effects of cell-derived hexameric Aβ by itself, but its capacity to aggravate amyloid deposition and induce cytotoxicity when there is Aβ to seed at disposal.

## Supplementary Information

Below is the link to the electronic supplementary material.

Supplementary file1 (XLSX 10 kb). Supplementary Table.S1. Title: Clinical analyses performed on the patients used in this study. Description: Non-AD (1258 and 1506), pre-clinical AD (1523, 1268 and 1556), and symptomatic AD (1272, 1329 and 1633) patients were monitored for memory impairments by the Mini-Mental State Examination (MMSE). Their CSF was collected for measurement of the two biomarkers of AD; Aβ and Tau. PET imaging using amyloid and/or Tau specific ligands was conducted in patients where the measurements exceeded pathological threshold. +=positive, N/A=non acquired

Supplementary file2 (PDF 3053 kb). Supplementary Fig.S1. Title: Detection of the ~28kDa assemblies by the anti-Aβ clone 6E10 primary antibody. Description: Cell lysates and media of CHO cells were processed as in Fig.1. Detection with the 6E10 clone targeting the N-terminus of human Aβ revealed the same ~28kDa bands as W0-2 when cells are expressing either C42 or C99, reinforcing the specific detection of Aβ in these assemblies. Dashed lines indicate that proteins were run on the same gel, but lanes are not contiguous. Supplementary Fig.S2. Title: Detection of the isolated ~28kDa assemblies by the anti-Aβ42, but not anti-Aβ40 or anti-APP-C-ter primary antibodies. Description: Dot blotting on the isolated ~28kDa assemblies revealed they are composed of the Aβ42 isoform. Synthetic preparations of monomeric Aβ40 and Aβ42 were used as positive controls. Combined with the observed size, we identify the assemblies of interest as Aβ42 hexamers. The absence of detection with the anti-APP-C-ter antibody was confirmed on the isolated assemblies, with C99-expressing cell lysates used as positive control. Dashed lines indicate that proteins were loaded on the same membrane, but image was readjusted. Supplementary Fig.S3. Title: Presenilins 1 and 2 protein levels. Description: Protein levels of PS1 and PS2 were monitored by Western blotting in SH-SY5Y wild-type (WT), scrambled (S) and knockdown (KD) cell lines. Quantification was performed on ImageJ (N=3 independent experiments) using α-tubulin as intra-experiment loading controls. Supplementary Fig.S4. Title: Absence of recognition of the ~28kDa assemblies present in EVs by the anti-APP-C-ter primary antibody. Description: Extracellular vesicles (EVs) isolated from the media of cultured PS1-S, PS1-KD, PS2-S, PS2-KD and PS2-R cells were monitored by Western blotting with the anti-APP-C-ter antibody. The C99 fragment (~10kDa) is recognized but not the ~28kDa assemblies, confirming they are formed by association of Aβ only. S=scrambled, KD=knockdown, R=rescued. Supplementary Fig.S5. Title: Presence of ~28kDa assemblies in the cerebrospinal fluid of human AD patients. Description: Hexameric-like Aβ assemblies were identified in the cerebrospinal fluid (CSF) of AD patients by Western blotting. Long, saturated exposures are represented in complement to Fig.5c for a better appreciation of the ~28kDa bands observed with the anti-Aβ (W0-2) antibody (left panel), which are not recognized by the anti-APP-C-ter antibody (right panel). sAPP=soluble APP. Pre-cl.=preclinical. Sympto.=symptomatic

## Data Availability

All datasets generated and analyzed during this study are included in this published article and its supplementary information files. Materials are available upon request.

## Abbreviations

Aβ: β-Amyloid
AD: Alzheimer’s disease
AICD: APP intracellular domain
APP: Amyloid precursor protein
CHO: Chinese hamster ovary
CRISPR: Clustered regularly interspaced short palindromic repeats
Cryo-EM: Cryo-electron microscopy
CSF: Cerebrospinal fluid
CTF: C-terminal fragment
HEK: Human embryonic kidney
KD: *knockdown*
MCI: Mild cognitive impairment
PET: Positron-emission tomography
*PSEN*: Presenilin (gene)
PS: Presenilin (protein)
R: Rescued
S: Scrambled
sAPP: Soluble APP
ThT: Thioflavin T
WT: Wild-type
