# Supplementary material for: Mechanism of Cellular Formation and In Vivo Seeding Effects of Hexameric β-Amyloid Assemblies
Source: Mol Neurobiol. 2021 Oct 4;58(12):6647–69. doi: 10.1007/s12035-021-02567-8 (PMC8639606; doi:10.1007/s12035-021-02567-8)

Supplementary Fig.S1

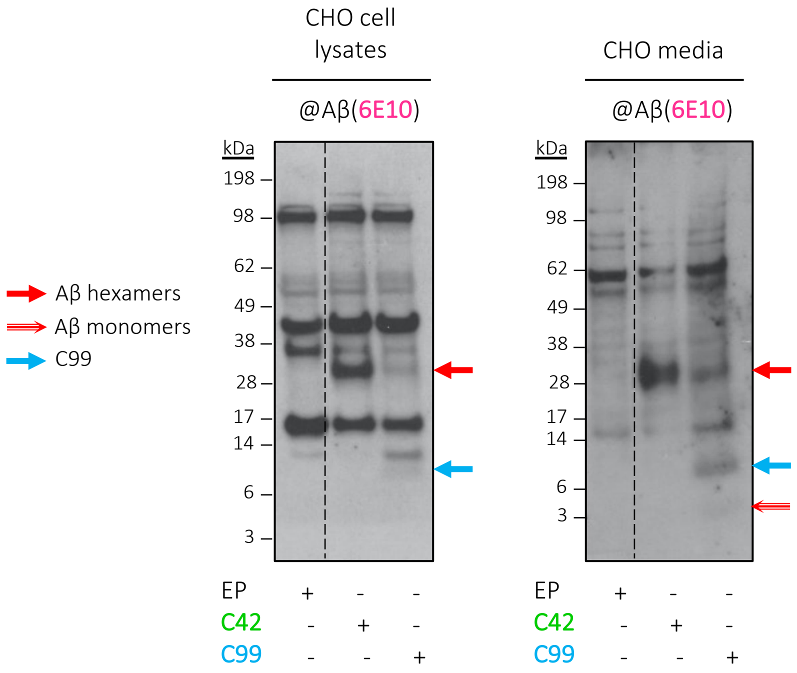

Supplementary Fig.S2

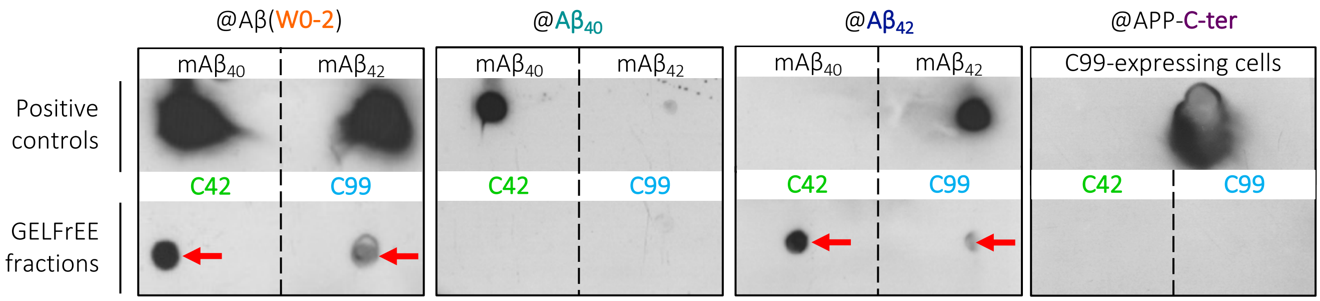

Supplementary Fig.S3

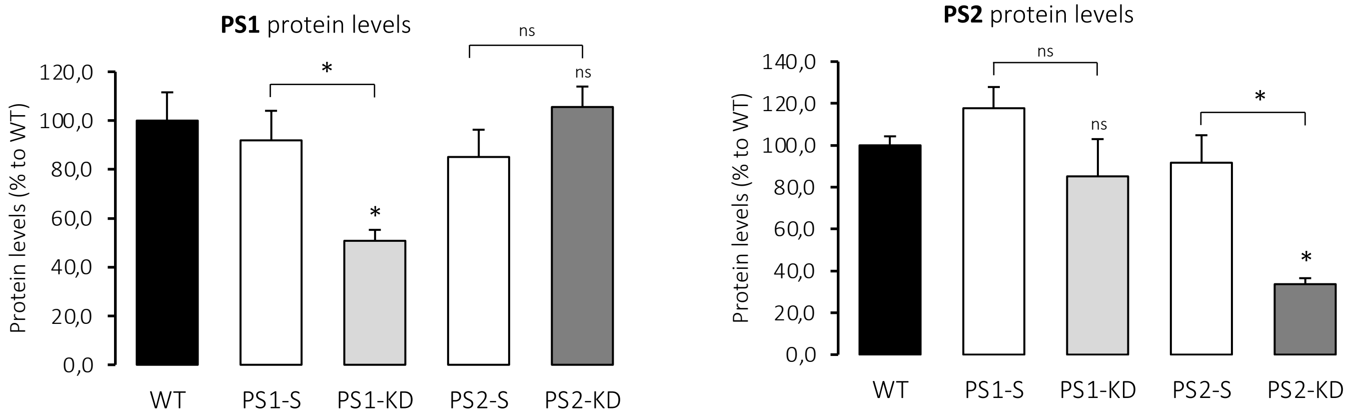

Supplementary Fig.S4

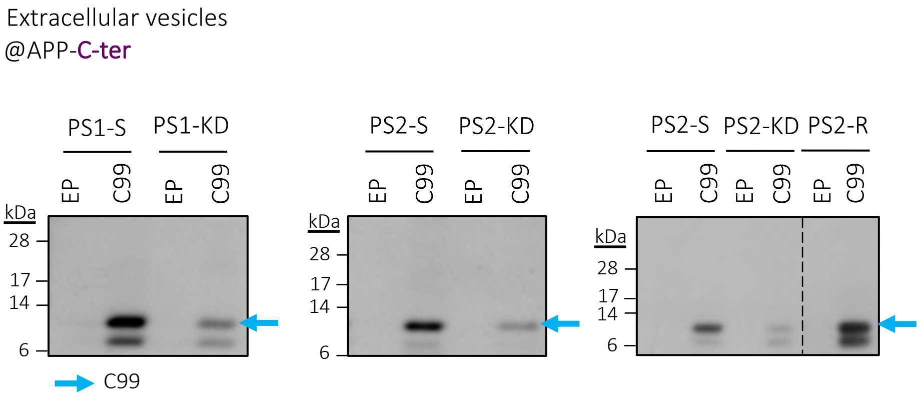

Supplementary Fig.S5

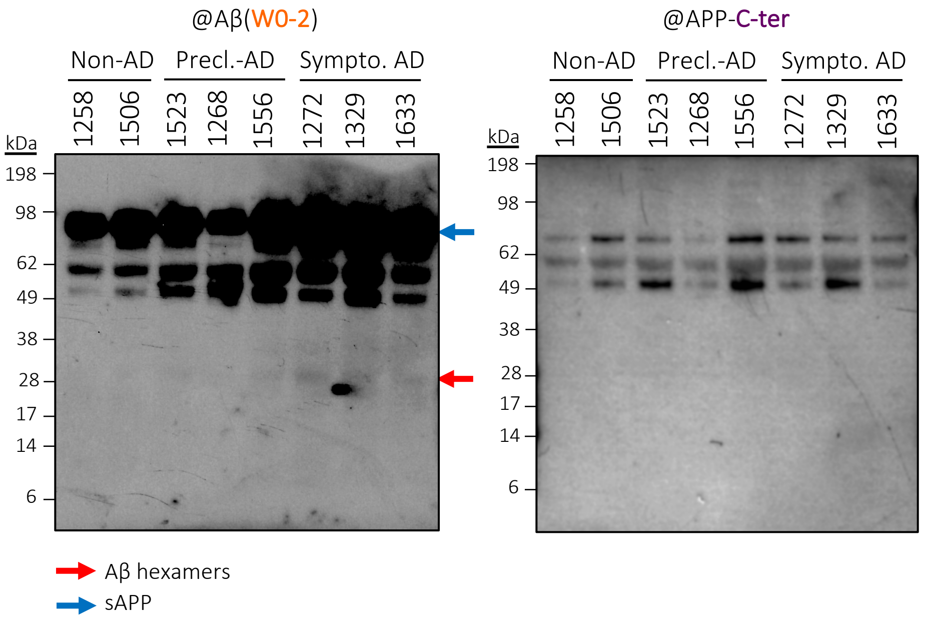

Supplement: Supplementary file 2 — Supplementary file2 (PDF 3053 kb). Supplementary Fig.S1. Title: Detection of the ~28kDa assemblies by the anti-Aβ clone 6E10 primary antibody. Description: Cell lysates and media of CHO cells were processed as in Fig.1. Detection with the 6E10 clone targeting the N-terminus of human Aβ revealed the same ~28kDa bands as W0-2 when cells are expressing either C42 or C99, reinforcing the specific detection of Aβ in these assemblies. Dashed lines indicate that proteins were run on the same gel, but lanes are not contiguous. Supplementary Fig.S2. Title: Detection of the isolated ~28kDa assemblies by the anti-Aβ42, but not anti-Aβ40 or anti-APP-C-ter primary antibodies. Description: Dot blotting on the isolated ~28kDa assemblies revealed they are composed of the Aβ42 isoform. Synthetic preparations of monomeric Aβ40 and Aβ42 were used as positive controls. Combined with the observed size, we identify the assemblies of interest as Aβ42 hexamers. The absence of detection with the anti-APP-C-ter antibody was confirmed on the isolated assemblies, with C99-expressing cell lysates used as positive control. Dashed lines indicate that proteins were loaded on the same membrane, but image was readjusted. Supplementary Fig.S3. Title: Presenilins 1 and 2 protein levels. Description: Protein levels of PS1 and PS2 were monitored by Western blotting in SH-SY5Y wild-type (WT), scrambled (S) and knockdown (KD) cell lines. Quantification was performed on ImageJ (N=3 independent experiments) using α-tubulin as intra-experiment loading controls. Supplementary Fig.S4. Title: Absence of recognition of the ~28kDa assemblies present in EVs by the anti-APP-C-ter primary antibody. Description: Extracellular vesicles (EVs) isolated from the media of cultured PS1-S, PS1-KD, PS2-S, PS2-KD and PS2-R cells were monitored by Western blotting with the anti-APP-C-ter antibody. The C99 fragment (~10kDa) is recognized but not the ~28kDa assemblies, confirming they are formed by association of Aβ only. S=scra [file 12035_2021_2567_MOESM2_ESM.pdf]
